# Alterations of matrix metalloproteinases in the healthy elderly with increased risk of prodromal Alzheimer's disease

**DOI:** 10.1186/alzrt44

**Published:** 2010-06-24

**Authors:** Erik Stomrud, Maria Björkqvist, Sabina Janciauskiene, Lennart Minthon, Oskar Hansson

**Affiliations:** 1 Clinical Memory Research Unit, Department of Clinical Sciences Malmö, Lund University; SUS, 205 02 Malmö, Sweden; 2 Neuropsychiatric Clinic, Skåne University Hospital; 205 02 Malmö, Sweden; 3 Neuronal Survival Unit, Department of Experimental Medical Science, Lund University; Wallenberg Neuroscience Center, BMC A10, Sölvegatan 17, Lund, Sweden; 4 Wallenberg Laboratory, Department of Clinical Sciences Malmö, Lund University; SUS, 205 02 Malmö, Sweden

## Abstract

**Introduction:**

Matrix metalloproteinases (MMP) are believed to be involved in the pathologic processes behind Alzheimer's disease (AD). In this study, we aimed to examine the cerebrospinal fluid (CSF) levels of MMPs and tissue inhibitors of metalloproteinase-1 (TIMP-1) in individuals with AD dementia and cognitively healthy elderly individuals, and to investigate their relationship with established CSF biomarkers for Alzheimer's disease.

**Methods:**

CSF was collected from 38 individuals with AD dementia and 34 cognitively healthy elderly individuals. The CSF was analyzed for MMP-1, MMP-3, MMP-9, TIMP-1, β-amyloid_1-42 _(Aβ42), total tau protein (T-tau) and phosphorylated tau protein (P-tau). MMP/TIMP-1 ratios were calculated. *APOE *genotype was determined for the participants.

**Results:**

AD patients had higher MMP-9/TIMP-1 ratios and lower TIMP-1 levels compared to cognitively healthy individuals. In AD patients, the MMP-9/TIMP-1 ratio correlated with CSF T-tau, a marker of neurodegeneration. Interestingly, the cognitively healthy individuals with risk markers for future AD, i.e. AD-supportive CSF biomarker levels of T-tau, P-tau and Aβ42 or the presence of the *APOE *ε4 allele, had higher CSF MMP-3 and MMP-9 levels and higher CSF MMP-3/TIMP-1 ratios compared to the healthy individuals without risk markers. The CSF levels of MMP-3 and -9 in the control group also correlated with the CSF T-tau and P-tau levels.

**Conclusions:**

This study indicates that MMP-3 and MMP-9 might be involved in early pathogenesis of AD and that MMPs could be associated with neuronal degeneration and formation of neurofibrillary tangles even prior to development of overt cognitive dysfunction.

## Introduction

Most cases of dementia are caused by Alzheimer's disease (AD), which is characterized by progressive accumulation of senile plaques, containing β-amyloid (Aβ), and neurofibrillary tangles, containing hyperphosphorylated tau [1]. This process probably starts many years before the typical clinical symptoms of AD appear. However, the underlying pathologic mechanisms in AD are still to a large extent unknown and the target of extensive research. There is increasing evidence indicating that matrix metalloproteinases (MMPs) may play an important but complex role in the pathology behind neurodegenerative disorders [2-4]. MMPs are zinc- and calcium-dependent endopeptidases, several of which are produced by neurons and glial cells. MMPs can be further divided into gelatinases (such as MMP-9), stromelysins (such as MMP-3), collagenases (such as MMP-1) and membrane-type MMPs (MT-MMP) [2,3]. Their activity is determined through the induction of transcription by inflammatory mediators, through post-translational modification by free radicals or cytokines and through inhibitory proteins such as tissue inhibitors of metalloproteinases (TIMPs) [3]. The different TIMPs often have inhibitory effects on most MMPs. However, they usually have a predisposition to one or a few MMPs, for example the inhibitory effect of TIMP-1 is primarily directed towards MMP-9 [2]. The tasks and effects of MMPs and TIMPs are complex, and the same MMP can have directly opposite effects on the brain depending on the situation, location, and time point in which it is being expressed. Their beneficial effects include neurogenesis, angiogenesis, myelinogenesis, axonal growth, and apoptosis inhibition, whereas examples of detrimental effects are apoptosis induction, blood brain barrier disruption and demyelination [2,3,5].

In AD, the expressions of MMP-3 and MMP-9 are elevated in the brain and are located around neurofibrillary tangles and amyloid plaques [6-8]. The activity of MMPs might be associated with the metabolism of Aβ, because Aβ has been found to induce the expression of MMPs by both astrocytes and neurons [9-12]. Moreover, MMP-3 and MMP-9 can cleave and degrade Aβ fibrils [8,13]. Recently, it has been suggested that MMP-9 expression in the hippocampus is involved in Aβ-induced cognitive dysfunction [10]. These findings together point to the need to increase our understanding of the role of MMPs in AD and their relation to other AD-related markers *in vivo*.

Investigation of markers in cerebrospinal fluid (CSF) is a valuable method to study pathologic processes in the brain. So far, the best validated CSF biomarkers for AD are low Aβ42 levels, high total tau protein (T-tau) levels and high levels of phosphorylated tau protein (P-tau). These biomarkers may also predict future AD dementia with acceptable accuracy in individuals with mild cognitive impairment (MCI) and they appear to be altered already in preclinical stages [14-20]. Apart from these biomarkers, the presence of the apolipoprotein E (*APOE*) ε4 allele is another well-established risk factor for the development of AD dementia [1].

The aim of this study was to investigate MMP-1, MMP-3, MMP-9 and TIMP-1 in the CSF of AD patients and healthy elderly controls, and their relation with the established CSF biomarkers Aβ42, T-tau and P-tau as well as the *APOE *genotype.

## Materials and methods

### Study population

The study population consisted of individuals with AD and healthy elderly individuals, who were all recruited at the Department of Neuropsychiatry at Malmö, Skåne University Hospital, Sweden. All individuals with AD were patients who had been referred to the clinic due to cognitive decline and had undergone a clinical, routine investigation. Patients with AD fulfiling the *Diagnostic and Statistical Manual of Mental Disorders *(*DSM*)-IIIR criteria for dementia [21] and the criteria for probable AD defined by the National Institute of Neurological and Communicative Disorders and Stroke and the Alzheimer's Disease and Related Disorders Association (NINCDS-ADRDA) [22] were eligible for the study.

The healthy elderly individuals were collected from a clinical control group with four years of cognitive follow-up and were summoned for an additional cognitive assessment and subsequent CSF collection. There is no clear, universal definition of the clinical characteristics of a cognitively healthy elderly individual. In the present study the cognitively healthy individuals were not allowed to fulfill criteria for dementia [21] or mild cognitive impairment [23] after extensive clinical and cognitive assessments. In order to additionally decrease the presence of possible early-stage cognitive impairment in the group, a mini mental state examination (MMSE) score of 27 points or more was required. This score is supported by several large-scale, community-based, normative studies that have reported mean MMSE values of 25 to 28 for individuals between 60 and 85 years of age, depending on age and educational level [24-26]. As these studies might have included some individuals with minor impairments in cognitive functions, the MMSE cut-off score in the present study was set slightly higher than the previously reported community-based mean values. In the present study the cognitive assessment also included the Alzheimer's Disease Assessment Scale (ADAS-cog 85 points), clock test, cube copying test and A Quick Test of cognitive speed (AQT). These results were taken into consideration in the decision whether dementia or MCI diagnosis criteria were fulfilled at the time of inclusion.

The study was approved by the Regional Ethics Committee at Lund University. All participants gave their consent to participate in the study.

### Study investigations

All participants in the study had their *APOE *genotype determined through blood testing. CSF collection was performed with the patient in a sitting position. After disposal of the first 1 ml of CSF, the next 10 ml were obtained from the L3/L4 or L4/L5 interspaces and collected in polypropylene tubes. The samples were centrifuged at 2,000 g at 4°C for 10 minutes to eliminate cells and other insoluble material, and were then immediately frozen and stored at -80°C pending biochemical analyses, without being thawed or refrozen. Cell count was performed on the CSF samples and no sample contained more that 500 erythrocytes/μl.

The CSF samples were analyzed for T-tau, tau protein phosphorylated at threonine 181 (P-tau) and Aβ42. In the AD patients, CSF T-tau concentration was determined using a sandwich ELISA (Innotest^® ^hTAU-Ag, Innogenetics, Ghent, Belgium) specifically constructed to measure all tau isoforms irrespective of phosphorylation status, as previously described [27]. CSF P-tau levels were determined using sandwich ELISA (Innotest^® ^PHOSPHO-TAU_(181P)_, Innogenetics, Ghent, Belgium). CSF Aβ_1-42 _levels were determined using a sandwich ELISA (Innotest^® ^β-amyloid (1-42), Innogenetics, Gent, Belgium) specifically constructed to measure Aβ containing both the 1^st ^and 42^nd ^amino acid, as previously described [28]. In the healthy elderly individuals analysis was performed with xMAP technology using the INNO-BIA AlzBio3 kit (Innogenetics, Ghent, Belgium) and the same batch of reagents [29]. Results from the Luminex xMAP system were converted to ELISA levels based on previously published conversion factors [29]. The results are presented in ng/l.

The CSF MMP-1, MMP-3, MMP-9 and TIMP-1 levels were measured using commercially available immunoassays according to the instructions provided by the manufacturers 'Meso Scale Discovery' (Gaithersburg, MD, USA) and 'R&D System' (Minneapolis, MN, USA), respectively. For the MMP assays a 12-point standard curve was used. The detection limit of the assay was 0.008 ng/ml of human recombinant MMP-1, 0.04 ng/ml of human recombinant MMP-3, 0.24 ng/ml of human recombinant MMP-9, and 0.031 ng/ml of human recombinant TIMP-1 with an inter-assay variation of less than 10%. The results are presented in ng/ml. The ratios of MMP-1/TIMP-1, MMP-3/TIMP-1 and MMP-9/TIMP-1 were additionally calculated.

For subgroup analyses, the healthy elderly individuals were divided according to deviant CSF biomarker levels and according to presence of the *APOE *ε4 allele, which are established risk markers for future dementia. Cut-off levels to define deviant CSF biomarker levels were determined at a CSF Aβ42/P-tau ratio of less than 6.5 combined with CSF T-tau levels of more than 350 ng/l. These cut-off levels were chosen because this combination has predicted future AD with a sensitivity of 95% and a specificity of 87% in a large MCI study population [15].

### Statistics

Statistical analysis was performed using the PASW software (former SPSS software, version 17.0.1 for Windows, SPSS Inc., Chicago, IL, USA). Non-parametric tests were used because normal distribution could not be assumed in the groups. Spearman rank correlation coefficient (r_s_) was used to test the degree of correlation between CSF biomarkers, MMP and TIMP-1 levels as well as the influence of age. Mann-Whitney U test was used when one of the variables was dichotomized (group comparisons, presence of deviant CSF biomarker levels, *APOE *ε4 allele carrier and gender). Fisher's Exact Test was used if both variables were dichotomized (group comparisons of *APOE *ε4 allele carrier and gender). The level of significance was set to *P *< 0.05.

## Results

### Participant characteristics

The characteristics for the AD group and the group of healthy elderly individuals are presented in Table 1. The 38 AD patients had significantly higher CSF T-tau and CSF P-tau levels, lower CSF Aβ42 levels, lower MMSE scores and higher presence of the *APOE *ε4 allele compared with the 34 healthy elderly individuals. No difference in age or gender was observed between the groups.

**Table 1:** Characteristics for the AD group and the group of healthy elderly individuals

	AD	HC	Group difference
**Demographics**			
Number	38	34	
Age	76 ± 7	77 ± 8	ns
Gender (F/M)	23/15	24/10	ns
MMSE	19 ± 5	29 ± 1	*P *< 10^-12^
*APOE *ε4 heterozygote (homozygote)	63% (13%)	24% (3%)	*P *< 10^-4^
CSF**			
Aβ42	307 ± 91	647 ± 166	*P *< 10^-11^
T-tau	938 ± 542	399 ± 194	*P *< 10^-8^
P-tau	106 ± 55*	64 ± 28	*P *< 10^-4^
MMP-1	0.024 ± 0.016	0.021 ± 0.01	ns
MMP-3	0.716 ± 0.35	0.700 ± 0.36	ns
MMP-9	0.902 ± 0.39	0.859 ± 0.35	ns
TIMP-1	0.035 ± 11.7	0.041 ± 13.0	*P *< 0.05
MMP-1/TIMP-1	0.697 ± 0.35	0.564 ± 0.25	ns
MMP-3/TIMP-1	21.1 ± 9.7	18.1 ± 9.8	ns
MMP-9/TIMP-1	26.9 ± 11.9	22.7 ± 11.8	*P *< 0.05

Aβ42, β-amyloid_1-42_; AD, Alzheimer's disease; APOE, apolipoprotein E; CSF, cerebrospinal fluid; F, female; HC, healthy controls; M, male; MMP, matrix metalloproteinase; MMSE, mini mental state examination; TIMP, tissue inhibitor of metalloproteinase.

* n = 28. ** Levels are in ng/l for Aβ42, T-tau and P-tau and in ng/ml for the MMPs and TIMP-1.

### CSF MMPs and TIMP-1 levels in the AD patients

**Figure 1:**
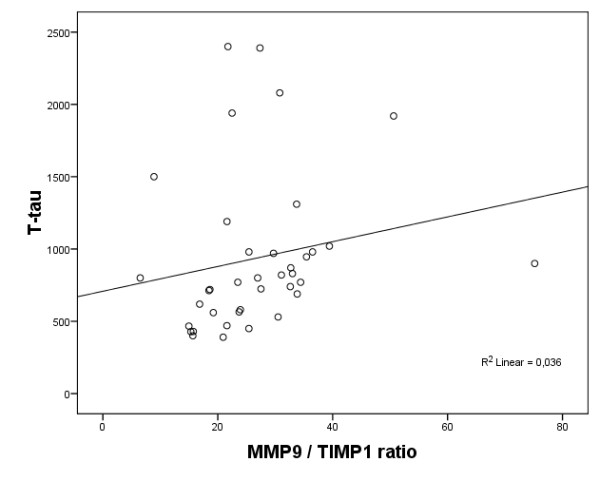
**Scatter plot of CSF MMP-9/TIMP-1 ratio and CSF T-tau levels in patients with Alzheimer's disease**. CSF T-tau levels are presented in ng/l. CSF, cerebrospinal fluid; MMP, matrix metalloproteinase; TIMP, tissue inhibitor of metalloproteinase.

**Figure 2:**
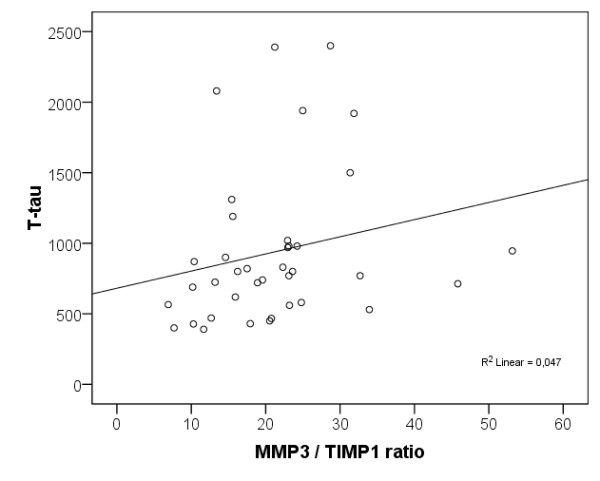
**Scatter plot of CSF MMP-3/TIMP-1 ratio and CSF T-tau levels in patients with Alzheimer's disease**. CSF T-tau levels are presented in ng/l. CSF, cerebrospinal fluid; MMP, matrix metalloproteinase; TIMP, tissue inhibitor of metalloproteinase.

The CSF MMP-9/TIMP-1 ratios were significantly higher and the CSF TIMP-1 levels were significantly lower in the AD patients compared with the healthy elderly individuals (*P *< 0.05; Table 1). Moreover, increased CSF T-tau levels correlated with high CSF MMP-9/TIMP-1 ratios (r_s _= 0.448, *P *< 0.01) and MMP-3/TIMP-1 ratios (r_s _= 0.351, *P *< 0.05) in the AD patients (Figures 1 and 2). *APOE *genotype, age and gender did not correlate with the CSF MMPs and TIMP-1 levels or with the CSF MMP/TIMP-1 ratio.

### CSF MMPs and TIMP-1 levels in the healthy elderly individuals

**Figure 3:**
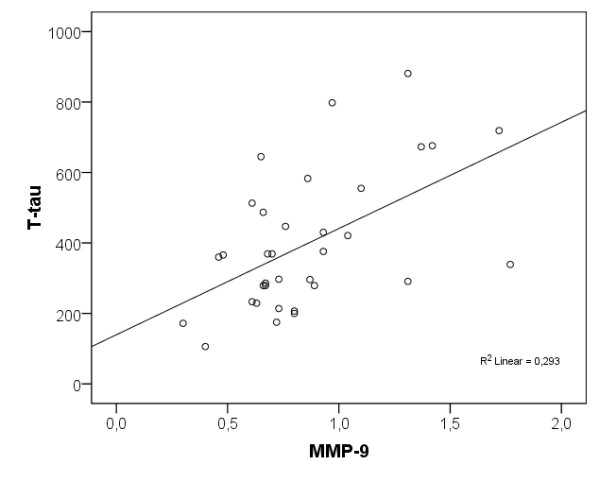
**Scatter plot of CSF MMP-9 levels and CSF T-tau levels in the cognitively healthy elderly individuals**. CSF MMP-9 levels are presented in ng/ml and CSF T-tau levels are presented in ng/l. CSF, cerebrospinal fluid; MMP, matrix metalloproteinase.

**Figure 4:**
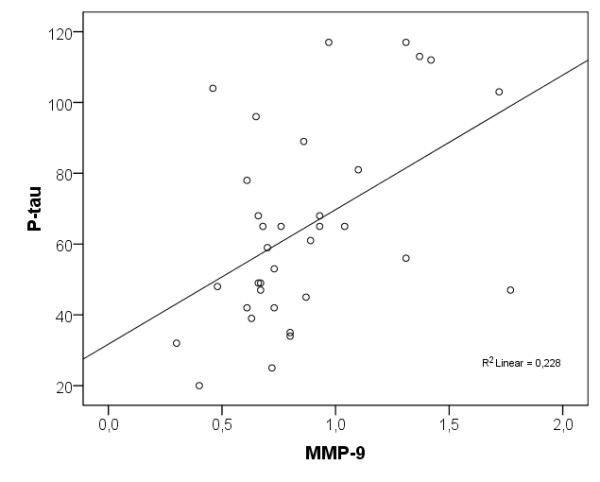
**Scatter plot of CSF MMP-9 levels and CSF P-tau levels in the cognitively healthy elderly individuals**. CSF MMP-9 levels are presented in ng/ml and CSF P-tau levels are presented in ng/l. CSF, cerebrospinal fluid; MMP, matrix metalloproteinase.

In the group of healthy elderly individuals, both higher levels of CSF MMP-9 and MMP-3 correlated with higher CSF T-tau levels (r_s _= 0.494, *P *< 0.01 and r_s _= 0.557, *P *< 0.001) and P-tau levels (r_s _= 0.435, *P *< 0.05 and r_s _= 0.554, *P *< 0.001; Figures 3 and 4). As seen in the AD participants, higher CSF MMP-3/TIMP-1 ratio correlated with higher CSF T-tau levels (r_s _= 0.352, *P *< 0.05) but in healthy elderly individuals the ratio also correlated with higher CSF P-tau levels (r_s _= 0.376, *P *< 0.05).

**Figure 5:**
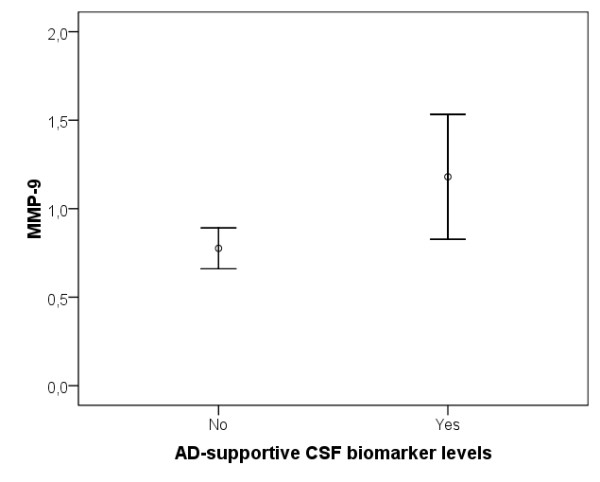
**Error plot of difference in CSF MMP-9 levels between the cognitively healthy elderly individuals with AD-indicative CSF biomarker levels (n = 7) compared with those with unaffected CSF biomarker levels (n = 27)**. CSF MMP-9 levels are presented in ng/ml. AD, Alzheimer's disease; CSF, cerebrospinal fluid; MMP, matrix metalloproteinase.

**Figure 6:**
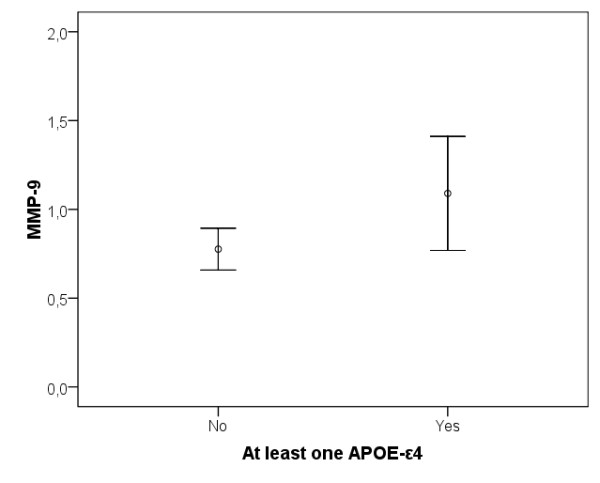
**Error plot of difference in MMP-9 levels between cognitively healthy elderly individuals with at least one *APOE *ε4 allele (n = 9) compared with those without the allele (n = 25)**. CSF MMP-9 levels are presented in ng/ml. CSF, cerebrospinal fluid; MMP, matrix metalloproteinase.

The seven healthy elderly individuals with an AD-supportive CSF biomarker pattern (CSF Aβ42/P-tau ratio <6.5 combined with CSF T-tau >350 ng/l [15]) had significantly higher levels of CSF MMP-9 (z = -2.37, *P *< 0.05; Figure 5) compared with the other 27 healthy elderly individuals. In addition, the nine *APOE *ε4 allele carriers had higher levels of CSF MMP-9 (z = -2.13, *P *< 0.05; Figure 6) and CSF MMP-3 (z = -2.23, *P *< 0.05) compared with the 25 non-carriers.

## Discussion

In the present study we show that AD patients have a higher MMP-9/TIMP-1 ratio and a lower TIMP-1 level in CSF compared with cognitively healthy elderly individuals and that the MMP-9/TIMP-1 ratio in AD patients correlates with CSF T-tau, a marker of neuronal degeneration. In the group of healthy elderly individuals we observed that the individuals with risk markers for possible future AD, that is AD-supportive CSF biomarkers (tau and Aβ42) or presence of the *APOE *ε4 allele, have higher CSF MMP-3 and MMP-9 levels and a higher CSF MMP-3/TIMP-1 ratio compared with the individuals without risk markers. In addition, the CSF MMP-3 and MMP-9 levels correlate with the CSF T-tau and P-tau levels in the elderly controls.

The findings of this study are in alignment with the elevated expression of MMP-3 and MMP-9 that have been observed in brain tissue of patients with established AD [7,8]. In particular, MMP-9 has been observed to have elevated expression in the hippocampus, around senile plaques and neurofibrillary tangles and in vessel walls [6,8], whereas MMP-3 is expressed primarily around plaques in the parietal lobes [7]. Hence, the expression of these MMPs is located in the brain regions and adjacent to histologic features that are closely related to AD. However, not all types of MMPs seem to be associated with AD pathogeneses. In the present study, MMP-1 (a collagenase) does not correlate with AD diagnosis or risk factors for future development of AD, a finding that is supported by a previous study that investigated the levels of different MMPs in plasma of AD patients [30].

The increased levels of several MMPs in the CSF of individuals with increased risk for AD, as observed in the current study, could be explained by several plausible mechanisms associated with AD pathology. In general, the CSF levels of MMPs are influenced by the production of MMPs by neurons and glial cells, the release of MMPs by inflammatory cells and extravasation of MMPs from peripheral blood. All of these mechanisms could be influenced by AD pathology. For example, increased levels of plasma MMP-9 have been observed in AD patients [30,31]. Both *in vitro *and *in vivo *studies have further suggested that the production of MMP-3 and MMP-9 are induced by AD-related proteins such as Aβ_1-40 _and Aβ_1-42 _[9-12]. Finally, pro-inflammatory molecules, which exist in and contribute to the AD neurodegenerative process, are inducers of MMP-3 and MMP-9 expression both locally in the brain and in inflammatory cells recruited from the peripheral circulation [2,12,32].

In the current study, CSF TIMP-1 levels were decreased and the MMP-9/TIMP-1 ratio was higher in AD patients when compared with healthy individuals. This could suggest the presence of an imbalance between MMP-9 and TIMP-1 in AD patients, which leads to a predominant MMP-9 activity in the brain. This idea is further supported by the association between high levels of CSF tau and high MMP-9/TIMP-1 ratios in the AD group.

A number of actions of MMPs and TIMPs, apart from the inflammatory response, have been closely linked to AD pathology. For example, MMP-9 has been shown to be able to cleave and degrade Aβ40 and aggregated Aβ42 fibrils both *in vitro *and *in vivo *[4,8,13]. In light of these possible AD protecting effects of MMP-9, it has been suggested that the increased MMP-9 levels observed in AD might be of the inactive form [2,8,12]. This could explain why Aβ still accumulates into plaques despite the increased levels of certain MMPs. It can further be noted that a specific MMP or TIMP has diverse effects on brain tissue depending on the situation, location and point in time of its expression and release [2,3]. An alternative interpretation of the increased MMP levels in AD could be that the detrimental activity of MMPs, which leads to brain damage, exceeds its beneficial brain protective activity. Interestingly, Mizoguchi and colleagues [10] have recently shown that Aβ-induced neurotoxicity *in vitro *as well as cognitive impairment *in vivo *is significantly alleviated by treatment with MMP inhibitors and significantly reduced in MMP-9 homozygous knockout mice, indicating that MMP-9 expression in the hippocampus might be involved in Aβ-induced cognitive dysfunction. Moreover, another study has shown increased activity of MMP-9 after stimulation with neurotoxic kainate in organotypic hippocampal cultures and reduced neuronal cell death after inhibition of MMP-9 [33]. Furthermore, in the same study, authors have shown that MMP-9 induces neuron death *in vitro*. Together these results indicate that MMP-9 might be involved in both Aβ-induced neuronal dysfunction as well as in excitotoxic cell death in the hippocampus.

The progressive neurodegeneration in AD precedes the decline in cognitive function and dementia diagnosis by decades [1]. The CSF biomarkers tau and Aβ are today well validated markers for AD pathology and they are associated with development of AD dementia in individuals with mild cognitive impairment [15-17]. Increasing evidence suggests that these CSF biomarkers indicate the presence of AD pathology also prior to the cognitive impairment, that is in cognitively unaffected elderly individuals [18-20,34,35]. In the present study certain MMPs and TIMP-1 levels were related to these CSF biomarkers. Interestingly, MMP-3 and MMP-9 were elevated in healthy elderly individuals with CSF biomarker levels implying an increased risk of future development of AD. In addition, increased CSF MMP-3 and MMP-9 levels in healthy elderly individuals correlated with the CSF levels of T-tau and P-tau. In contrast, no correlations were seen with CSF levels of Aβ42, which is surprising due to the possible relation between MMP-9 and amyloid pathology seen in animal models and the fact that Aβ42 is thought to be the first biomarker to be changed in preclinical AD [36]. However, a recent neuropathologic study reports that elevated MMP-9 activity correlates with Braak stage but not with NIA-Reagan diagnosis [37]. The major difference between Braak stage and the NIA-Reagan criteria is that the former only evaluates presence of tau pathology, whereas the latter evaluates presence of both tau and amyloid pathology [38,39]. In summary, our findings suggest that MMPs may be associated with AD pathology as well as with the presence of neuronal degeneration and formation of neurofibrillary tangles already in cognitively unaffected individuals.

Healthy elderly individuals with at least one *APOE *ε4 allele exhibit a three-fold increased risk of developing AD later on and are thereby more likely to have ongoing progressive neurodegenerative processes in the brain [1]. The higher CSF MMP-3 and MMP-9 levels seen in the healthy elderly individuals with at least one *APOE *ε4 allele in the present study, further supports a probable relation between MMPs and the presence of preclinical AD pathology. In alignment with this finding, Saarela and colleagues [40] have previously shown that the presence of the *APOE *ε4 allele together with a certain MMP-3 polymorphism increases the risk for developing AD in cognitively unaffected elderly individuals more than the presence of *APOE *ε4 allele alone [40].

A limitation of the study might be that CSF T-tau, P-tau and Aβ42 levels were measured with different methods in the AD patients compared with the healthy elderly individuals. The aim of the present study, however, was not to make group comparisons of these three CSF biomarkers. Instead, they were used to employ correlations with other markers within each group and to define 'individuals within the control group with increased risk for future AD'. The group difference in the CSF analysis method should therefore not influence the findings of the study. Moreover, it should be stated that the present data do not suggest MMP and TIMP-1 levels to be used for diagnostic discrimination. For this purpose, the overlap between the groups are too great and the discriminatory ability too low compared with currently accepted biomarkers such as CSF T-tau, P-tau and Aβ42. Similarly, the specificity of MMP and TIMP-1 levels to AD can not be evaluated in the present study because it was not designed to study other dementia disorders than AD. Another limitation could be that extensive neuropsychological testing was not performed on the control individuals and that cognitive follow-up currently only exists up to the time of inclusion. However, the cognitive assessments performed at and prior to the inclusion together with the extensive clinical assessment should have minimized the presence of early-stage cognitive impairment in the control group. Despite these efforts complete absence of preclinical cognitive impairment in the group can not be ensured.

## Conclusions

In the present study, the CSF MMP-9/TIMP-1 ratio was increased in AD patients, and correlated with the neuronal degeneration marker tau. More importantly, cognitively healthy elderly individuals, with increased risk of developing AD in the future, had elevated CSF MMP-3 and MMP-9 levels and an increased CSF MMP-3/TIMP-1 ratio, indicating that MMP-3 and MMP-9 might be involved in early pathogenesis of AD. Moreover, CSF levels of MMP-3 and MMP-9 correlated with both CSF T-tau and P-tau in elderly controls, suggesting that MMPs could be associated with neuronal degeneration and/or the formation of P-tau-containing neurofibrillary tangles in individuals who have not yet developed any overt cognitive dysfunction.

## Abbreviations

Aβ: β-amyloid; AD: Alzheimer's disease; ADAS-cog: Alzheimer's Disease Assessment Scale; APOE: apolipoprotein E; AQT: A Quick Test of cognitive speed; CSF: cerebrospinal fluid; ELISA: enzyme-linked immunosorbent assay; MCI: mild cognitive impairment; MMP: matrix metalloproteinase; MT-MMP: membrane-type MMP; P-tau: phosphorylated tau; MMSE: mini mental state examination; TIMP: tissue inhibitor of metalloproteinase; T-tau: total tau.

## Competing interests

The authors declare that they have no competing interests.

## Authors' contributions

ES participated in the design of the study, acquisition of data, statistical analysis and drafted the manuscript. MB performed the immunoassays. SJ performed the immunoassays. LM conceived the study. OH conceived the study, participated in the design of the study, performed the statistical analysis and drafted the manuscript All authors read and approved the final manuscript.
